# Stochastic and Heterogeneous Cancer Cell Migration: Experiment and Theory

**DOI:** 10.1038/s41598-019-52480-3

**Published:** 2019-11-08

**Authors:** Taejin Kwon, Ok-Seon Kwon, Hyuk-Jin Cha, Bong June Sung

**Affiliations:** 1 Department of Chemistry and Research Institute for Basic Science, Sogang University, Seoul, 04107 Republic of Korea; 2 Department of Life Sciences, Sogang University, Seoul, 04107 Republic of Korea; 3 College of Pharmacy, Seoul National University, Seoul, 08826 Republic of Korea

**Keywords:** Biophysical chemistry, Statistical mechanics, Biological physics

## Abstract

Cell migration, an essential process for normal cell development and cancer metastasis, differs from a simple random walk: the mean-square displacement (〈(Δ*r*)^2^(*t*)〉) of cells sometimes shows non-Fickian behavior, and the spatiotemporal correlation function (*G*(*r*, *t*)) of cells is often non-Gaussian. We find that this intriguing cell migration should be attributed to heterogeneity in a cell population, even one with a homogeneous genetic background. There are two limiting types of heterogeneity in a cell population: *cellular heterogeneity* and *temporal heterogeneity*. Cellular heterogeneity accounts for the cell-to-cell variation in migration capacity, while temporal heterogeneity arises from the temporal noise in the migration capacity of single cells. We illustrate that both cellular and temporal heterogeneity need to be taken into account simultaneously to elucidate cell migration. We investigate the two-dimensional migration of A549 lung cancer cells using time-lapse microscopy and find that the migration of A549 cells is Fickian but has a non-Gaussian spatiotemporal correlation. We find that when a theoretical model considers both cellular and temporal heterogeneity, the model reproduces all of the anomalous behaviors of cancer cell migration.

## Introduction

Cell migration is essential to normal cell development^1–3^, cancer metastasis^4–6^ and wound healing^7–9^. Developing a mathematical model for cell migration^10–15^ would help in understanding cell migration and designing new strategies to cure cancers and treat wounds. Because the trajectories of cells look quite similar to a random walk at certain timescales, most mathematical models are based on the diffusion equation and its equivalents such as a stochastic differential equation. Recent studies reported, however, that cell migration should be not only stochastic but also heterogeneous^16–18^: the cell-to-cell variation in migration capacity is significant, and/or a single cell undergoes temporal transitions in migration capacity. This heterogeneity makes the development of a mathematical model for cell migration tremendously challenging. In this study, we investigate the effects of heterogeneity on A549 lung cancer cell migration and compare various models to describe the heterogeneous trajectories of A549 cancer cells.

Heterogeneity is inherent in a cell population. The heterogeneity of a cancer cell population is significant even when the cancer cell line is derived from a single clone with the same genetic background^19–26^. Heterogeneity in a cell population may be categorized into two limiting cases: *cellular heterogeneity* and *temporal heterogeneity*. Cellular heterogeneity (population noise) accounts for time-independent cell-to-cell variation, while temporal heterogeneity (temporal noise) results from temporal fluctuations of single cells. Cellular and temporal heterogeneity are not mutually exclusive, as population and temporal noises may occur simultaneously in cell populations. Heterogeneity can result from a number of spatiotemporal factors such as different stages of the cell cycle^27^, circadian rhythm^28^ and the level of adenosine triphosphate (ATP)^29^ or Ca^2+^ ^30^ at an individual single cell level, which determine migration capacity. Therefore, an issue of fundamental importance is finding ways beyond population averaging to understand cell migration in terms of heterogeneity.

The diffusion equation, which describes the migration of particles, is based on two fundamental observations: (a) a particle is neither created nor destroyed, and (b) the flux of particles is proportional to a particle density gradient^31^. The diffusion equation leads to two important results: (1) the mean-square displacement (〈(Δ*r*)^2^(*t*)〉), a measure of how far the particle diffuses in a given time *t*, should be linearly proportional to time *t* at long times, i.e., $\langle {(\Delta r)}^{2}(t)\rangle  \sim {t}^{1}$, and (2) the particle displacement should follow Gaussian statistics due to the central limit theorem such that the spatiotemporal correlation function (*G*(*r*, *t*)) should be Gaussian. The concept of diffusion (and hence a random walk) has been employed to describe the cell migration. For example, a persistent random walk (PRW) model has been employed extensively to interpret and simulate cell migration^10,11,32–34^. Recent studies on cell migration showed, however, that cell migration is anomalous^12–15,35–38^: (1) cells sometimes showed non-Fickian behavior, i.e., $\langle {(\Delta r)}^{2}(t)\rangle  \sim {t}^{\alpha }$ with *α* ≠ 1, and (2) *G*(*r*, *t*) was non-Gaussian. We also find that A549 lung cancer cells migrate with a non-Gaussian *G*(*r*, *t*). This finding implies that the random walk model (and the conventional PRW model) would not hold for cells and that a new mathematical model would be required.

Previous studies illustrated that if one were to incorporate the cellular heterogeneity into the PRW model with different parameters for different cells, the non-Gaussian *G*(*r*, *t*) of HT1080 fibrosarcoma cells could be reproduced^15^. On the other hand, a single cell underwent temporal transitions between fast and slow states^36,39–42^. Metzner *et al*.^36^ proposed a statistical framework to model and analyze heterogeneous cell migration of the breast carcinoma cell line MDA-MB-231. They found that in the presence of such temporal heterogeneity, the spatiotemporal correlation function could be non-Gaussian, which a simple random walk model can not elucidate. Scientific questions arise: how cellular and temporal heterogeneity relate to the anomalous cell migration, and whether one can develop a mathematical model that combines both cellular and temporal heterogeneity. In this work, to compare with A549 lung cancer cells and answer these questions, we employ the following four different theoretical models: (1) homogeneous (HO) model without any type of heterogeneity, (2) cellular heterogeneity (CH) model with only cellular heterogeneity considered, (3) temporal heterogeneity (TH) model with only temporal heterogeneity and (4) cellular and temporal heterogeneity (CTH) model with both cellular and temporal heterogeneity. We find that only the CTH model may reproduce the experimental results for not only *G*(*r*, *t*) but also the spatiotemporal correlation function *g*_*i*_(*r*, *t*) of each single cell (not averaged over the population of cells) of A549 lung cancer cells, and that both cellular and temporal heterogeneity need to be taken into account to elucidate single-cell migration.

The rest of the paper is organized as follows. The experimental and cell tracking methods are described in the Materials and Methods section, the results are presented and discussed in the Results and Discussion section, and the conclusions are presented in the Conclusion section.

## Results and Discussion

### Migration of A549 cancer cells

**Figure 1:**
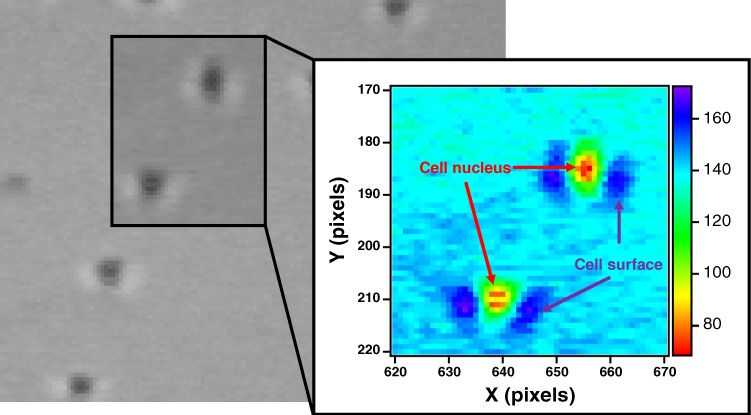
Image of A549 cells obtained by time-lapse microscope. (inset) Color code represents the brightness of pixels. The number of tracked A549 cells is 212.

**Figure 2:**
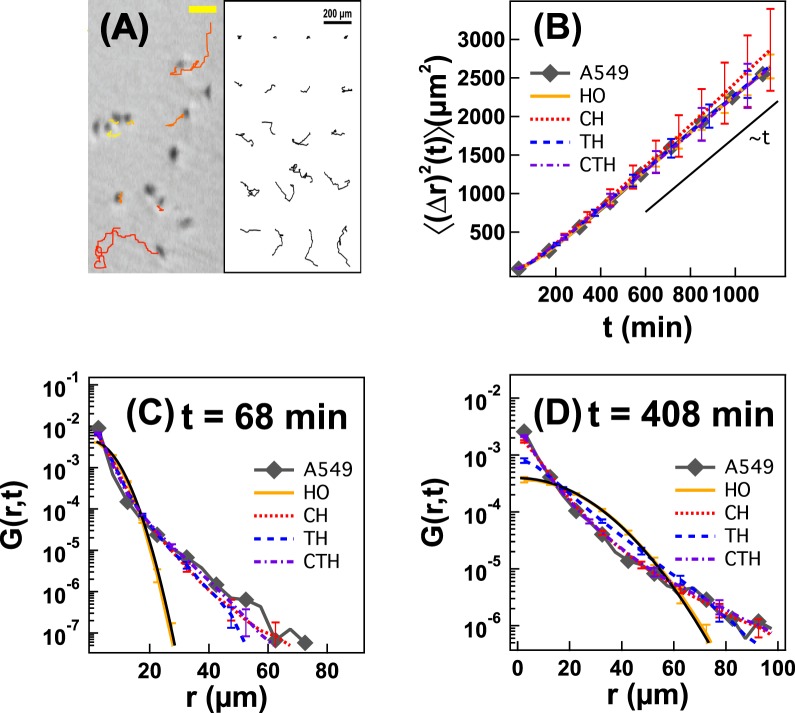
(**A**) Representative trajectories of A549 cells obtained from time-lapse microscopy. The yellow bar is 100 *μm* long in the figure. (**B**) 〈(Δ*r*)^2^(*t*)〉 averaged over all cells as function of *t*. *G*(*r,t*) averaged over all cells as function of *r* at (**C**) *t* = 68 min and (**D**) *t* = 408 min. Symbols represent the results obtained from the trajectories of A549 cells. Lines represent the results obtained from stochastic simulations based on the HO, CH, TH and CTH models. Black solid lines are Gaussian guidelines. Error bars in figure are standard deviations obtained from 50 simulations for each model.

We obtain the trajectories of A549 cancer cells by using time-lapse microscopy (as shown in Figs 1 and 2(A)). We estimate the mean velocity (${\vec{v}}_{i}(t)$) of each A549 cell during a Δ*t* = 34 min interval, i.e.,

1 $${\vec{v}}_{i}(t)=\frac{{\vec{r}}_{i}(t+\Delta t)-{\vec{r}}_{i}(t)}{\Delta t}.$$

Here, ${\overrightarrow{r}}_{i}(t)(=({\hat{x}}_{i},{\hat{y}}_{i}))$ denotes the position vector of the *i*th A549 cell at time *t*. Note that the time resolution is limited to 34 min in our experiment because the root of the mean-square displacement of A549 cells reaches the dimension (5 *μ*m) of one pixel only after 34 min. See more details in Supporting Information. We estimate the mean-square displacement 〈(Δ*r*)^2^(*t*)〉 as follows:

2 $$\langle {(\Delta r)}^{2}(t)\rangle =\frac{1}{N}\mathop{\sum }\limits_{i\mathrm{=1}}^{N}\,{\langle {\vec{r}}_{i}(t+t^{\prime} )-{\vec{r}}_{i}(t^{\prime} )\rangle }_{t^{\prime} }^{2},$$

where ${\langle \cdots \rangle }_{t^{\prime} }$ denotes a time average over different time origins *t*′ and *N* is the number of A549 cells. The integral form of the time average is ${\langle \cdots \rangle }_{t^{\prime} }=\frac{1}{\tau }{\int }_{0}^{\tau }\,\cdots dt^{\prime}$, where *τ* is the total measurement time in our experiment. Therefore, 〈(Δ*r*)^2^(*t*)〉 is a quantity averaged over both times and all cells. We also obtain the spatiotemporal correlation function *g*_*i*_(*r*, *t*) of each A549 cell as follows:

3 $${g}_{i}(r,t)={\langle \delta \{\vec{r}-[{\vec{r}}_{i}(t+t^{\prime} )-{\vec{r}}_{i}(t^{\prime} )]\}\rangle }_{t^{\prime} },$$

where *δ* is the Dirac delta function. One can construct a histogram and obtain *g*_*i*_(*r*, *t*) numerically by checking whether the displacement ($|{\vec{r}}_{i}(t+t^{\prime} )-{\vec{r}}_{i}(t^{\prime} )|$) of the *i* th cell from *t*′ to *t*′ + *t* lies between a certain range. Note that *g*_*i*_(*r*, *t*) is averaged not over cells but over different time origins *t*′. The physical meaning of 2*πrg*_*i*_(*r*, *t*) is the conditional probability that the *i* th A549 cell migrates a distance *r* during a time interval *t*. *G*(*r*, *t*) is then obtained by ensemble-averagin*g g*_*i*_(*r*, *t*) over all A549 cells in the population, i.e.,

4 $$G(r,t)=\frac{1}{N}\mathop{\sum }\limits_{i\mathrm{=1}}^{N}\,{g}_{i}(r,t).$$

Note that the physical meanings of *g*_*i*_(*r*, *t*) and *G*(*r*, *t*) are identical. While *G*(*r*, *t*) is a property averaged over the population of A549 cells, *g*_*i*_(*r*, *t*) is the property of each single A549 cell. If cells were to undergo a random walk and follow the diffusion equation, both *G*(*r*, *t*) and *g*_*i*_(*r*, *t*) would be Gaussian.

Our experiment shows that A549 cells undergo Fickian yet non-Gaussian cell migration. 〈(Δ*r*)^2^(*t*)〉 becomes linearly proportional to time *t* at long timescales, and A549 cells reach a Fickian regime (gray symbols in Fig. 2(B)). More interesting is that *G*(*r*, *t*) for A549 cells is non-Gaussian. As depicted in Fig. 2(C) and (D), all A549 cells have a non-Gaussian *G*(*r*, *t*) at *t* = 68 and 408 min, which results from the violation of the central limit theorem and is unexpected from the conventional PRW model. *t* = 68 min and *t* = 408 min represent time scales before and after persistent time (*P* = 78 min), respectively. Our migration results show that the cell migration of A549 cells is anomalous both before and after persistent time (*P* = 78 min).

**Figure 3:**
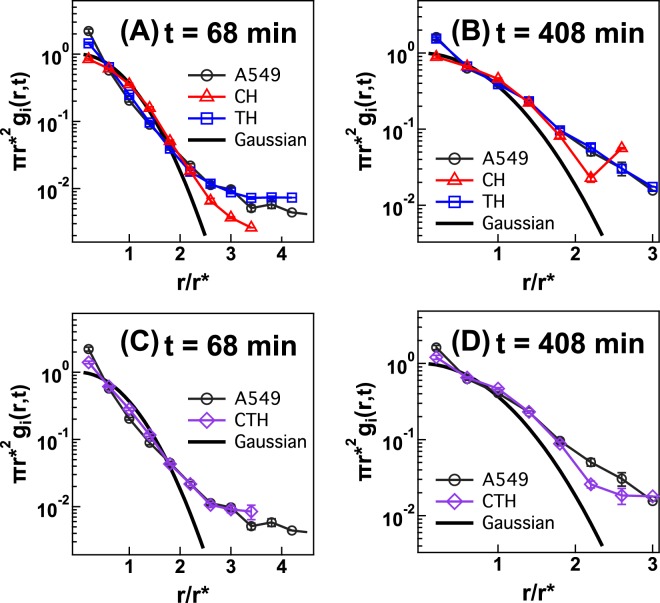
Rescaled spatiotemporal correlation functions (πr*^2^*g*_*i*_(*r, t*)) of individual A549 cells (black circles), which are not averaged over cell population. Rescaled *g*_*i*_(*r*, *t*)’s are binned on the x-axis. Markers and error bars are the mean and standard error of the mean of binned data. Black solid lines are Gaussian guidelines. The simulation results for rescaled *g*_*i*_(*r*, *t*) obtained from the CH (red triangles) and TH (blue squares) models at (**A**) *t* = 68 min and (**B**) *t* = 408 min are presented. The simulation results for rescaled *g*_*i*_(*r, t*) obtained from the CTH (purple diamonds) model at (**C**) *t* = 68 min and (**D**) *t* = 408 min are also presented.  𝑟^∗^(≡√⟨(Δ𝑟)^2^(𝑡)⟩) is root-mean-square displacement of each cell trajectory at a given time *t*.

The spatiotemporal correlation function *g*_*i*_(*r*, *t*) of each single A549 cell is also non-Gaussian at *t* = 68 min and *t* = 408 min. Figure 3 depicts rescaled *g*_*i*_(*r*, *t*) of A549 cells. Here, r* denotes the root-mean-square displacement of each single A549 cell at time t, i.e., ${r}^{\ast }=\sqrt{\langle {(\Delta r)}^{2}(t)\rangle }$. One can compare the *g*_*i*_(*r*, *t*) of A549 cells (black circles in Fig. 3(A)) with a solid Gaussian guideline and find that the rescaled *g*_*i*_(*r*, *t*) of A549 cells is far from being Gaussian at *t* = 68 min. Some A549 cells migrate a very long distance, up to *r*/r* = 4 at *t* = 68 min, which is different from a simple random walk that follows Gaussian statistics.

Non-Gaussian migration has also been observed in complex systems such as nanoparticle diffusion in polymeric materials^43–46^. Previous studies took heterogeneity into account and suggested theoretical models to elucidate the non-Gaussian migration^47–49^. Chubynsky and his coworkers proposed a theory based on a stochastic differential equation where the diffusion coefficient of a particle also diffused, i.e., *diffusing diffusivity*^49^. In this approach, the temporal heterogeneity is incorporated, and the particle is supposed to undergo a temporal transition in the migration state. Other studies proposed that complex systems would consist of domains of different mobility: particles in fast domains migrate quickly and particles in slow domains migrate slowly, which is similar to the notion of cellular heterogeneity in the sense that different cells migrate with different migration capacity.

### Theoretical models for anomalous cell migration

To investigate how cellular and temporal heterogeneity affect cell migration, we consider four different theoretical models, as discussed below. We perform stochastic simulations based on those four theoretical models and compare the simulation results with the experimental results for A549 cells. The parameters required for stochastic simulations are obtained from the experiment on A549 cells. A summary of four models and parameters is described in the Table 1. In this study, the simulation time of all models is 1292 min, which is the same as the experimental time.

**Table 1:** A summary of the HO, CH, TH, and CTH models.

	HO model	CH model	TH model	CTH model
Parameter	*S*, *P*	*S*_*i*_, *P*_*i*_	*A*_*th*_	*A*_*cth*_
How to get parameters	fitting to 〈(Δ*r*)^2^(*t*)〉	*S*_*i*_: mean speed of each cell *P*_*i*_: decay time of $\langle {\overrightarrow{v}}_{i}(t){\overrightarrow{v}}_{i}\mathrm{(0)}\rangle /\langle {\overrightarrow{v}}_{i}^{2}\mathrm{(0)}\rangle$	fitting to 〈(Δ*r*)^2^(*t*)〉	fitting to 〈(Δ*r*)^2^(*t*)〉
*G*(*r*, *t*)	Gaussian	non-Gaussian	non-Gaussian	non-Gaussian
*g*_*i*_(*r*, *t*)	Gaussian w/o cell-to-cell variation	Gaussian with cell-to-cell variation	non-Gaussian w/o cell-to-cell variation	non-gaussian with cell-to-cell variation

### HO model without heterogeneity

The PRW model has been employed extensively to interpret cell migration and is based on a stochastic differential equation as follows^11,15^:

5 $$\frac{d{v}_{i}(t)}{dt}=-\frac{1}{P}{v}_{i}+\frac{S}{\sqrt{P}}\hat{w},$$

where *v*_*i*_(*t*) is the velocity of the *i* th cell. $\hat{w}$ represents the random noise of the Wiener process, and *P* and *S* denote the persistent time and the magnitude of the mean velocity of cells, respectively. The term $\frac{S}{\sqrt{P}}$ in front of the random noise corresponds to the magnitude of noise in HO model. One can find the relation between the magnitude (*S*) of the mean velocity and the magnitude of the noise by deriving the mean-squared velocity from the Eq. 5 ^11^. We employ an integrator developed by Bussi *et al*.^50^ to perform the numerical simulations of the Eq. 5. The integration time step is 0.01 min. If the cell-to-cell variation in cell migration was absent, all of the cells would have identical values of *P* and *S*. For this homogeneous cell migration, the mean-square displacement 〈(Δ*r*)^2^(*t*)〉 in two dimensions is derived readily from the above stochastic differential equation as follows:

6 $$\langle {(\Delta r)}^{2}(t)\rangle =2{S}^{2}{P}^{2}({e}^{-\frac{t}{P}}+\frac{t}{P}-1)+4{\sigma }_{err}^{2},$$

where *σ*_*err*_ represents the magnitude of the localization error, which may arise due to spatial resolution in the experiment. Note that the first term of the Eq. 6 converges to zero as *t* → 0, while the second term is a constant. The first term corresponds to the mean-square displacement of the *true position* of A549 cells. In the experiment, however, our position measurement of the A549 cell is unavoidably limited by the localization error due to the spatiotemporal resolution such that the localization error (the second term) should be incorporated into the Eq. 6^13,15,51^.

We obtain the values of *P*, *S*, and *σ*_*err*_ by fitting 〈(Δ*r*)^2^(*t*)〉 of A549 cells with a sampling time of 34 min to Eq. 6 using the least squares method (See more details and Fig. S2 in Supporting Information): *S* = 0.125 *μ*m/min, *P* = 78 min, and *σ*_*err*_ = 1.66 *μ*m. Then, we perform stochastic simulations using the above HO model (the Eq. 5) and values of *P*, *S*, and *σ*_*err*_. The localization error is added to the simulated trajectories to compare to the experiment as follows:

7 $$\hat{x}(t)=x(t)+{\sigma }_{err}\cdot W.$$

**Figure 4:**
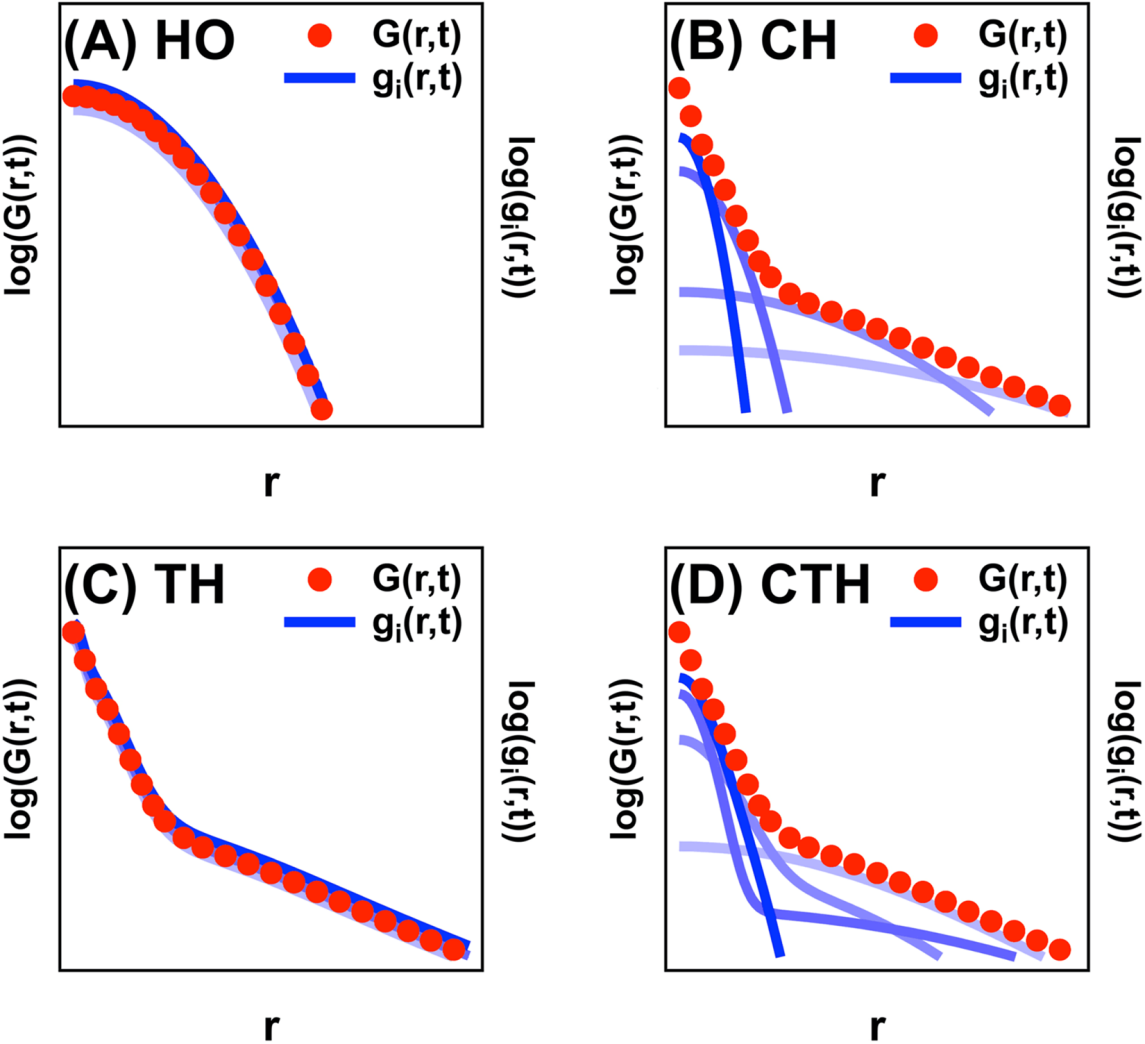
Schematic figures of the four different theoretical models employed in this study. Red symbols represent *G*(*r*, *t*) while blue lines represent *g*_*i*_(*r*, *t*)’s of individual single cells of each model. (**A**) HO model, where *G*(*r*, *t*) = *g*_*i*_(*r*, *t*) and both *G*(*r*, *t*) and *g*_*i*_(*r*, *t*) are Gaussian. Since all cells are assumed to show identical dynamic behaviors, *g*_*i*_(*r*, *t*)’s collapse onto one another. (**B**) In the CH model, *G*(*r*, *t*) ≠ *g*_*i*_(*r*, *t*), and *G*(*r*, *t*) is non-Gaussian, while *g*_*i*_(*r*, *t*) is Gaussian. Since cellular heterogeneity is introduced in the CH model, *g*_*i*_(*r*, *t*)’s are different from each other. (**C**) In the TH model, *G*(*r*, *t*) = *g*_*i*_(*r*, *t*), and both *G*(*r*, *t*) and *g*_*i*_(*r*, *t*) are non-Gaussian. Because cellular heterogeneity is not considered in the TH model, *g*_*i*_(*r*, *t*)’s of individual cells collapse onto each other. (**D**) In the CTH model with both cellular and temporal heterogeneity, *G*(*r*, *t*) ≠ *g*_*i*_(*r*, *t*), and both *G*(*r*, *t*) and *g*_*i*_(*r*, *t*) are non-Gaussian. In the CTH model, cellular heterogeneity is incorporated such that *g*_*i*_(*r*, *t*)’s of individual cells are different from each other.

Here, *x*(*t*) is the trajectory obtained from simulations, *W* is white Gaussian noise of unit variance, and $\hat{x}$(*t*) is the simulated trajectories with the localization error included. Even though *x*(*t*) is the true position in simulations obtained by solving the Eq. 5, we need to compare the mean-square displacement of $\hat{x}$(*t*) with 〈(Δ*r*)^2^(*t*)〉 of A549 cells because our position measurement for A549 cells is also limited by the localization error. We incorporate the localization error into the trajectories from all 4 models. As depicted schematically in Fig. 4(A), the HO model predicts that both *G*(*r*, *t*) and *g*_*i*_(*r*, *t*) are identical to each other and are Gaussian.

### CH model with cellular heterogeneity

Wu *et al*. proposed a theoretical approach based on the PRW model to incorporate cellular heterogeneity^15^. In this approach, they assumed that the cell-to-cell variation in migration capacity should be large enough that each cell would possess its own values of *P* and *S*. The different values are assigned to the persistent time (*P*_*i*_) and the magnitude of mean velocity (*S*_*i*_) of the *i* th cell. Then, the *i* th cell obeys the following stochastic differential equation:

8 $$\frac{d{v}_{i}(t)}{dt}=-\frac{1}{{P}_{i}}{v}_{i}+\frac{{S}_{i}}{\sqrt{{P}_{i}}}\hat{w}.$$

In this CH model, the cell-to-cell variation in cell migration is incorporated into the distributions of *P*_*i*_ and *S*_*i*_.

To obtain the value of *P*_*i*_ for the *i* th A549 cell from time-lapse microscopy, we set the value of *P*_*i*_ to a decay time when the normalized velocity autocorrelation function ($\langle {\overrightarrow{v}}_{i}(t){\overrightarrow{v}}_{i}(0)\rangle /\langle {\overrightarrow{v}}_{i}^{2}(0)\rangle$) of the *i* th cell equals 1/e (Fig. S3). Note that $\langle {\overrightarrow{v}}_{i}(t){\overrightarrow{v}}_{i}(0)\rangle /\langle {\overrightarrow{v}}_{i}^{2}(0)\rangle$ of an individual A549 cell is not exponential but *P*_*i*_ is still required to test the CH model. Therefore, we obtain a representative value for the persistent time *P*_*i*_ by employing the equation $\langle {\overrightarrow{v}}_{i}(t){\overrightarrow{v}}_{i}(0)\rangle /\langle {\overrightarrow{v}}_{i}^{2}(0)\rangle =1/e$ using linear interpolation. Then, we round up *P*_*i*_ to the multiples of the sampling time (34 min). Considering the spatial and temporal resolution in our experiment, the sampling time of 34 min is a sufficiently short timescale in this study. The values of the cell speed and continuous and discrete persistent times of individual cells are reported for comparison in the Supporting Information (Fig. S4). As discussed below, the CH model with the discrete persistent times obtained in this study successfully reproduces the 〈(Δ*r*)^2^(*t*)〉 and *G*(*r*, *t*) of A549 cells. The value of *S*_*i*_ is also obtained from the experiment by estimating the average magnitude of the *i* th A549 cell’s magnitude of mean velocity. The values of the cell speed and continuous and discrete persistent time of individual cells are reported in the Supporting Information (Fig. S4). We perform stochastic simulations by using Eq. 8 and the values of *P*_*i*_ and *S*_*i*_ obtained from A549 cells, which are conducted using the same integrator that was used in the HO model. The integration time step is 0.01 min.

In the CH model, each individual cell obeys the above stochastic differential equation based on the PRW model such that the *g*_*i*_(*r*, *t*) of each cell should be Gaussian. *g*_*i*_(*r*, *t*)’s are all Gaussian but have different values for variance for different cells. However, $G(r,t)(\equiv \frac{1}{N}{\sum }_{i\mathrm{=1}}^{N}\,{g}_{i}(r,t))$ becomes non-Gaussian because *G*(*r*, *t*) is obtained by averaging over all cells (Fig. 4(B)).

### TH model with temporal heterogeneity

If a cell were to undergo correlated transitions between different migration states, the conventional PRW model (with fixed values of *P* and *S* for each cell) would not describe the cell migration properly. The conventional PRW model assumes that the cells stay in a single migration state (characterized by *P* and *S*). If the temporal heterogeneity were significant, the values of *P* and *S* of the cell would change with time such that the spatiotemporal correlation function *g*_*i*_(*r*, *t*) of the individual cell would be non-Gaussian, which is different from the CH model. Therefore, the conventional PRW stochastic differential equation (such as Eqs. 5 and 8) cannot be used.

To scrutinize whether the temporal heterogeneity alone could result in anomalous cell migration, we need to consider a case in which each cell undergoes the correlated transition in migration states but the population of cells is still homogeneous. This means that all of the cells in the population possess the same degree of the temporal heterogeneity such that *G*(*r*, *t*) = *g*_*i*_(*r*, *t*) for any *i* (Fig. 4(C)). To realize such systems with temporal heterogeneity and *cellular homogeneity*, we propose the following stochastic differential equation (Eq. 9) and perform stochastic simulations in which the magnitude (*β*(*t*)) of the random noise changes with time *t*.

9 $$d{\vec{r}}_{i}(t+dt)=\frac{d{\vec{r}}_{i}(t)}{{A}_{th}}+\beta (t)\vec{\xi },$$

Here, $d{\vec{r}}_{i}(t)$ is the displacement vector of the *i* th cell at time *t*. $\vec{\xi }$ is a unit vector with random orientation, which only determines the direction. $\beta (t)\vec{\xi }$ orients uniformly on the plane from 0 to 2 *π* and is uncorrelated with that of previous steps a*t t* − *dt. β*(*t*) is the magnitude of the random noise and relates to the distance that the cell migrates during *dt*.

In order to simulate the TH model, one needs to obtain *β*(*t*) and *A*_*th*_ as discussed below. We sample *β*(*t*) from the distribution 2*πrG*(*r*, *t* = *P*) of A549 cells using an inverse transform sampling method because the physical meaning of 2*πrG*(*r*, *t* = *P*) is the probability distribution function that the cell would migrate by *r* during the time interval of *P*. Here, *P* is 68 min due to a sampling time of 34 min (instead of P = 78 min obtained from fitting 〈(Δ*r*)^2^(*t*)〉). As discussed below, TH model using the discrete persistent time also reproduces successfully 〈(Δ*r*)^2^(*t*)〉 and *G*(*r*, *t*) of A549 cells. In order to sample *β*(*t*), we calculate the cumulative distribution function of 2*πrG*(*r*, *t* = *P*) and invert that function. The inverted cumulative distribution function transforms a random variable (uniformly sampled between 0 and 1) to a random variable *β*(*t*). *β*(*t*) changes with time *t* such that each individual cell undergoes temporal transitions in migration states, which is not possible in the HO and CH models (See the Supporting Information for details). Note that *β*(*t*) is not a fitting parameter but a stochastic variable sampled from 2*πrG*(*r*, *t* = *P*) of A549 cell trajectories. *A*_*th*_ is a parameter that indicates how persistent the cell migration would be. In the OU process, for example, *A*_*th*_ corresponds to *exp*(*dt*/*P*). We obtain the value of *A*_*th*_ ( = 2.5) by fitting and reproducing the mean-square displacement of A549 cells (See more details and Fig. S6(A) in the Supporting Information.) We perform simulations of Eq. 9 using the Monte Carlo method. The integration time step, *dt*, is the same as the persistent time *P*.

As depicted schematically in Fig. 4(C), the TH model predicts that *G*(*r*, *t*) = *g*_*i*_(*r*, *t*) but both *G*(*r*, *t*) and *g*_*i*_(*r*, *t*) are non-Gaussian. This is because all cells undergo the correlated transitions between different migration states in an identical fashion (Eq. 9) via *β*(*t*).

### CTH model with both cellular and temporal Heterogeneity

In the last theoretical model, we aim to combine both cellular and temporal heterogeneity by modifying the above TH model. In this model, each cell may undergo temporal changes in its migration state of different degrees for different cells. To fulfill both temporal and cellular heterogeneity in the numerical simulation, we propose the following stochastic differential equation and perform stochastic simulations:

10 $$d{\overrightarrow{r}}_{i}(t+dt)=\frac{d{\overrightarrow{r}}_{i}(t)}{{A}_{cth}}+{\beta }_{i}(t)\overrightarrow{\xi },$$

where the value of *P*_*i*_ is obtained from the *i* th A549 cell and *β*_*i*_ (the magnitude of the temporal noise of the *i* th cell) is sampled randomly from 2*πrg*_*i*_(*r*, *t* = *P*_*i*_) of the *i* th A549 cell instead of 2*πrG*(*r*, *t* = *P*) (As mentioned above, CTH model uses discrete *P*_*i*_).

In order to simulate the CTH model, one needs to obtain *β*_*i*_(*t*) and *A*_*cth*_. When sampling *β*_*i*_(*t*), we calculate the cumulative distribution function of 2*πrg*_*i*_(*r*, *t* = *P*_*i*_) and invert that function. The inverted cumulative distribution function transforms a random variable (uniformly sampled between 0 and 1) to a random variable *β*_*i*_(*t*). *β*_*i*_ changes with time *t* such that each cell may undergo a temporal transition between migration states (See in Fig. S5 in the Supporting Information). At the same time, *β*_*i*_ is sampled from *g*_*i*_(*r*, *t* = *P*_*i*_) of each cell, thus ensuring that the temporal transition varies with each cell. *β*_*i*_(*t*) is not a fitting parameter but a stochastic variable sampled from 2*πrg*_*i*_(*r*, *t* = *P*_*i*_). Therefore, one should obtain *P*_*i*_ and *g*_*i*_(*r*, *t* = *P*_*i*_) of each cell. *A*_*cth*_ = 2.4 is used in the CTH model to reproduce the averaged mean-square displacement of A549 cells (See Fig. S6(B) in Supporting Information). Note that *A*_*cth*_ is identical for all cells and does not depend on each cell. But, *P*_*i*_ and *g*_*i*_(*r*, *t*) of cells in the CTH model are heterogeneous, because *β*_*i*_ is sampled from 2*πrg*_*i*_(*r*, *t* = *P*_*i*_) of each cell (See Figs S8(D) and S9 in Supporting Information). We also conduct simulations of the CTH model using the Monte Carlo method. In the CTH model, the integration time step is different for different cells because each cell has its own persistent time. The integration time step of the *i* th cell is the persistent time of the *i* th cell (*P*_*i*_). The experimental results for *g*_*i*_(*r*, *t* = *P*_*i*_) obtained from A549 cells are reproduced by the stochastic simulations based on the CTH model.

### Comparison of A549 cells and theoretical models

#### Average cell migration

We perform stochastic simulations based on the four different theoretical models and compare the results to the experimental results for A549 cells. Even the HO model, into which we do not incorporate any heterogeneity, reproduces the experimental result for 〈(Δ*r*)^2^(*t*)〉 (Fig. 2(B)). This success of the HO model for 〈(Δ*r*)^2^(*t*)〉 has been well known. The other three models (with cellular and/or temporal heterogeneity considered) also reproduce 〈(Δ*r*)^2^(*t*)〉 successfully. To investigate how consistent the four theoretical models would be with the experiment for 〈(Δ*r*)^2^(*t*)〉, we estimate the p-values of the 4 models. We compare 〈(Δ*r*)^2^(*t*)〉 of A549 cells with 〈(Δ*r*)^2^(*t*)〉 obtained from 50 simulations for each model and calculate *χ*-squared values^52^. The p-values (excluding the data point of 〈(Δ*r*)^2^(*t*)〉 at *t* = 34 min) are 0.4338 (HO model), 0.9742 (CH model), 1 (TH model), and 0.9792 (CTH model). The p-values of all of the models is more than 0.2 after 68 min, suggesting that 〈(Δ*r*)^2^(*t*)〉 from the simulations is consistent with the experiments for A549 cells. This indicates that 〈(Δ*r*)^2^(*t*)〉 is not a suitable physical quantity when one tries to investigate the effects of heterogeneity in the cell population on the cell migration.

The HO model fails, however, to reproduce the *G*(*r*, *t*) of A549 cells at both short and long timescales. As shown in Fig. 2(C) and (D), the *G*(*r*, *t*) obtained from the HO model is Gaussian at both *t* = 68 and 408 min. This is because the HO model (the conventional PRW model) should be, in principle, based on the random walk model such that *G*(*r*, *t*) from the HO model is expected to be Gaussian. On the other hand, the *G*(*r*, *t*) of A549 cells is non-Gaussian at *t* = 68 and *t* = 408 min. All of the other theoretical models (with heterogeneity considered to some extent) succeed in reproducing the non-Gaussian *G*(*r*, *t*) at both short and long timescales. Wu *et al*. also showed that the CH model could explain the non-Gaussian *G*(*r*, *t*) of HT1080 fibrosarcoma cells^15^. Metzner *et al*. illustrated that when they employed temporal heterogeneity, they could elucidate non-Gaussian *G*(*r*, *t*) of MBA-MB-231 cells^36^. Our CTH model with both cellular and temporal heterogeneity also captures such non-Gaussian cell migration.

We estimate the root mean-squared logarithmic error (RMSLE) for *G*(*r*, *t*) and rescaled *g*_*i*_(*r*, *t*) of four models as follows,

11 $$RMSLE=\sqrt{\frac{1}{{N}_{t}}\mathop{\sum }\limits_{i=1}^{{N}_{t}}\,{\{log({c}_{i}+1)-log({\hat{c}}_{i}+1)\}}^{2}},$$

where *c* denotes either *G*(*r*, *t*) or rescaled *g*_*i*_(*r*, *t*) of four models, and $\hat{c}$ denotes the corresponding values from the experiment. *N*_*t*_ is the number of data points in Fig. 2 and 3. As shown in Table 2, the CTH model shows the smallest value of RMSLE for *G*(*r*, *t* = 68 *min*) and *G*(*r*, *t* = 408 *min*). In case of the rescaled *g*_*i*_(*r*, *t*), the CH model has the largest RMSLE value while the RMSLE values of the TH and CTH model are comparable for the rescaled *g*_*i*_(*r*, *t* = 68 *min*). In case of rescaled *g*_*i*_(*r*, *t* = 408 *min*), the TH model produces a better result than the CTH and CH models. As shall be discussed in the following section, however, the TH model fails to capture the cellular heterogeneity of the A549 cell migration while the CTH model successfully reflects the cellular heterogeneity (See Fig. S8 in Supporting Information).

**Table 2:** Root mean-squared logarithmic error (RMSLE) of 4 models for *G*(*r*, *t*) and rescaled *gi*(*r*, *t*).

y	HO model	CH model	TH model	CTH model
$G(r,\,t=68\,min)$	0.0004976	0.0003021	0.0002649	0.0002018
*G*(*r*, *t* = 408 *min*)	0.0002218	0.00008106	0.0001748	0.00004009
Rescaled *gi*(*r*, *t* = 68 *min*)		0.0717	0.03452	0.03754
Rescaled *g*_*i*_(*r*, *t* = 408 *min*)		0.05117	0.005978	0.02768

**Figure 5:**
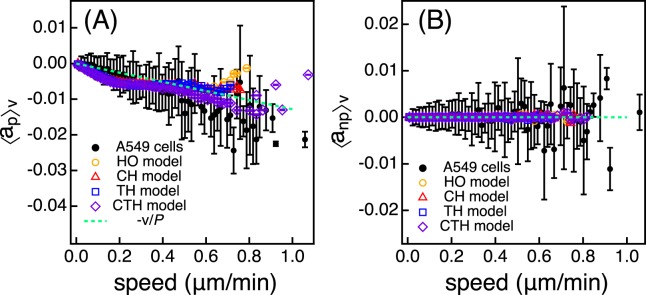
(**A**) 〈a_*p*_〉_*v*_ and (B) 〈*a*_*np*_〉_*v*_ as a function of cell speed for the HO (yellow empty circles), CH (red triangles), TH (blue squares), and CTH models (purple diamonds). Black filled circles represent A549 cells. Note that error bars in this figure indicate the standard errors of the mean. The dotted lines are guidelines with slopes of (**A**) -*v*/*P* and (**B**) zero.

We estimate the magnitude of the deterministic term and the noise term by calculating the magnitude of the mean deviation of acceleration ($\overrightarrow{a}(t)=(\overrightarrow{v}(t+dt)-\overrightarrow{v}(t))/dt$)^13,51,53–55^ to see whether the deterministic term and the noise term would be dependent on the cell speed. First, we calculate the component (${a}_{p}\equiv \overrightarrow{a}(t)\cdot \overrightarrow{v}(t)/|\overrightarrow{v}(t)|$) of the acceleration along the cell velocity ($\overrightarrow{v}(t)$) and then estimate the magnitude (〈*a*_*p*_〉_*v*_) of the conditional average of the component *a*_*p*_ for a given $v=|\overrightarrow{v}(t)|$ and the magnitude (|*a*_*p*_−〈*a*_*p*_〉_*v*_|) of the mean deviation of the component. From the component (*a*_*np*_) of the acceleration orthogonal to the cell velocity, we estimate the conditional average (〈*a*_*np*_〉_*v*_) of the component *a*_*np*_ for a given *v* and the magnitude (|*a*_*np*_−〈*a*_*np*_〉_*v*_|) of mean deviation of the component of acceleration orthogonal to cell velocity. Figure 5 shows that 〈*a*_*p*_〉_*v*_ of A549 cells and all models follows −*v*/*P* and that 〈*a*_*np*_〉_*v*_ of A549 cells and all models is zero. Previous studies also showed that 〈*a*_*p*_〉_*v*_ followed −*v*/*P* and 〈*a*_*np*_〉_*v*_ was zero regardless of speed^13,51^, which is consistent with our results.

**Figure 6:**
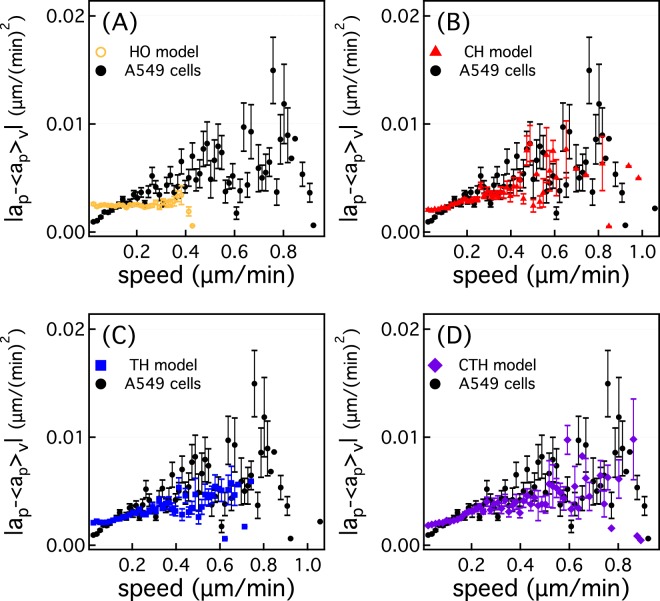
Magnitude of the mean deviation (|*a*_*p*_−〈*a*_*p*_〉_*v*_|) of the component of acceleration along the cell velocity as a function of cell speed for the (**A**) HO (yellow empty circles), (**B**) CH (red triangles), (**C**) TH (blue squares), and (**D**) CTH models (purple diamonds). Black filled circles represent A549 cells. Note that error bars in this figure indicate the standard error of the mean.

Figure 6(A) shows that the magnitude of noise of the HO model is uniform regardless of the speed, as expected. On the other hand, the magnitude of noise of A549 cells is dependent on the cell speed, unlike the HO model. As shown in Fig. 6(B), (C), and (D), the CH, TH and CTH models well reproduce |*a*_*p*_−〈*a*_*p*_〉_*v*_| of A549 cells. In the case of the CH model, according to Eq. 8, the magnitude of the noise term is determined by $\frac{{S}_{i}}{\sqrt{{P}_{i}}}$. Therefore, each cell owns its own values for *S*_*i*_ and *P*_*i*_ such that cells with larger speed may have large |*a*_*p*_−〈*a*_*p*_〉_*v*_|, which makes the magnitude of noise of the CH model dependent on the speed. On the other hand, for the TH model, the magnitude of noise term of the TH model is determined by *β*(*t*). If a cell were to have a large speed, the cell is supposed to migrate by a large distance during a given time and *β*(*t*) may be large, too. This leads to the dependence of |*a*_*p*_−〈*a*_*p*_〉_*v*_| on speed. Similarly, for the CTH model, the magnitude of noise term of the CTH model is determined by *β*_*i*_(*t*). CTH model takes into account the cellular heterogeneity such that a faster cell is likely to have a larger value for *β*_*i*_(*t*). This also leads to the dependence of |*a*_*p*_−〈*a*_*p*_〉_*v*_| on speed. We also investigate the component of the acceleration orthogonal to the cell velocity (Fig. S10 in Supporting Information). Even for the orthogonal component, the CH, TH and CTH models reproduce the dependence of the noise on the cell speed.

Pedersen *et al*.^51^ showed that 〈*a*_*p*_〉_*v*_ and |*a*_*p*_−〈*a*_*p*_〉_*v*_| were dependent on the sampling time and the localization error. Our results could be also affected by the choice of sampling time and the magnitude of the localization error. Especially, |*a*_*p*_−〈*a*_*p*_〉_*v*_| at a zero speed in all models is not zero due to the localization error. In addition, because we obtain the localization error from A549 cell data of the sampling time of 34 min, |*a*_*p*_−〈*a*_*p*_〉_*v*_| of numerical simulations at time scales shorter than the sampling time is overestimated than that of A549 cells. Please note that, however, the CH, TH, and CTH models well describe the dependence of |*a*_*p*_−〈*a*_*p*_〉_*v*_| on the cell speed of A549 cells after about 0.2 *μ*m/min.

### Individual cell migration

The stochastic simulations based on the CH model result in Gaussian *g*_*i*_(*r*, *t*) at short timescales of *t* = 68 min and long timescales of *t* = 408 min, which differs from that of A549 cells. Because we rescale both *g*_*i*_(*r*, *t*) and *r* in Fig. 3(A), rescaled *g*_*i*_(*r*, *t*)'s of different values of *P*_*i*_ and *S*_*i*_ in the CH model collapse onto a single Gaussian curve (red symbols in Fig. 3(A)). The CH model can reproduce *G*(*r*, *t*) but fails to reproduce *g*_*i*_(*r*, *t*). Because the CH model is based on the conventional PRW model but has different values of *P*_*i*_ and *S*_*i*_, each trajectory from the CH model should result in a Gaussian *g*_*i*_(*r*, *t*). This is different from A549 cells because the trajectory of each single A549 cell leads to non-Gaussian *g*_*i*_(*r*, *t*) at short timescales of *t* = 68 min and long timescales of *t* = 408 min (Fig. 3(B)).

On the other hand, the TH model captures the non-Gaussian behavior of the *g*_*i*_(*r*, *t*) of A549 cells. As shown in Fig. 3(A) and (B), rescaled *g*_*i*_(*r*, *t*)’s of the TH model reproduce those of A549 cells. However, the TH model fails to capture the cell-to-cell variation in *g*_*i*_(*r*, *t*)’s of A549 cells. As shown in Fig. S7, the 25th A549 cell migrates more than 40 *μ*m at *t* = 408 min, while other A549 cells do not. The TH model does not reflect such cell-to-cell variation, and *g*_*i*_(*r*, *t*)’s obtained from the TH model all collapse to a single curve (Fig. S8(C)).

The CTH model (which combines both cellular and temporal heterogeneity) reproduces not only rescaled *g*_*i*_(*r*, *t*)’s at both short and long timescales (Fig. 3(C,D)) but also the cell-to-cell variation in *g*_*i*_(*r*, *t*). Rescaled *g*_*i*_(*r*, *t*)’s from the CTH model are non-Gaussian and overlap with those of A549 cells at *t* = 68 and 408 min. As shown in Fig. S8(D), the *g*_*i*_(*r*, *t*) obtained using parameters extracted from the 25*th* cell is clearly distinguished from the *g*_*i*_(*r*, *t*) obtained using parameters extracted from the 212*th* cell, thus indicating that the CTH model captures well the cell-to-cell variation in *g*_*i*_(*r*, *t*).

The failure of the HO and TH models could be expected because those two models do not consider the cellular heterogeneity in a A549 cell population. Figure S9 in the Supporting Information depicts the distribution of the persistent time (*P*_*i*_) obtained from the normalized velocity autocorrelation functions $\langle {\overrightarrow{v}}_{i}(t){\overrightarrow{v}}_{i}(0)\rangle /\langle {\overrightarrow{v}}_{i}^{2}(0)\rangle$ of A549 cells and stochastic simulations. Because all of the cells in the TH model should undergo migration homogeneously, the *P*_*i*_ values of these cells does not differ from one another much. On the other hand, *P*_*i*_ of A549 cells has a broad distribution, which can be quantitatively reproduced only in the CH and CTH models. Our comparison of *G*(*r*, *t*) and *g*_*i*_(*r*, *t*) between the experiment and simulations illustrates clearly that either cellular heterogeneity or temporal heterogeneity may explain *G*(*r*, *t*) averaged over the A549 cells population. However, only when we incorporate both cellular and temporal heterogeneity together into the CTH model can we elucidate both individual (*g*_*i*_(*r*, *t*)) and ensemble (*G*(*r*, *t*)) properties of the cell migration.

Previous studies for cell migration have also reported heterogeneous cell migration^15,36^. For example, Metzner *et al*.^36^ proposed a statistical framework for modeling and analyzing heterogeneous cell migration, and showed that a cell migration model based on an autoregressive process of first order (AR-1 process) described the anomalous cell migration of MDA-MB-231 cells successfully. However, the cell migration model based on an AR-1 process still assumed that the migration of cells under the same condition would be determined by one single distribution, which corresponds to the TH model in this paper. On the other hands, the CTH model assumes that each cell would have different migration capacities by considering *P*_*i*_ and *g*_*i*_(*r*, *t*) of an individual cell obtained in the experiment.

## Conclusion

We investigate the migration of A549 cells that possess intermediate characteristics between epithelial and mesenchymal states. A549 cells exhibit Fickian diffusion with $\langle {(\Delta r)}^{2}(t)\rangle  \sim {t}^{1}$. However, the spatiotemporal correlation function (*G*(*r*, *t*)) averaged over the population of A549 cells is non-Gaussian at both short and long timescales. More interesting is that the spatiotemporal correlation function of individual cells (*g*_*i*_(*r*, *t*)) is non-Gaussian at short and long timescales.

We find that such anomalous cell migration should be attributed to the heterogeneity in a A549 cell population. To elucidate the origin of the anomalous migration of A549 cells, we employ four different theoretical models and carry out stochastic simulations. The HO model does not take any type of heterogeneity into account: all of the cells are assumed to migrate with identical persistent times and magnitudes of the mean cell velocity. On the other hand, other theoretical models (CH, TH and CTH models) consider the cellular and/or temporal heterogeneity. The CH model considers the cellular heterogeneity with different values of persistent time (*P*_*i*_) and magnitude of the mean cell velocity (*S*_*i*_) for different cells. We obtain the values of *P*_*i*_ and *S*_*i*_ from the trajectory of each A549 cell, which are used again for the stochastic simulations based on Eq. 8.

In case of the TH model, the temporal heterogeneity, where cells may undergo transition between different migration states, is taken into account. In this case, the spatiotemporal correlation function (*g*_*i*_(*r*, *t*)) of a single cell could be non-Gaussian. We assume in the TH model that all of the cells would undergo the temporal transition to the same extent such that the population of cells could be homogeneous. The quantity *β*(*t*) (which is related to the magnitude of the mean velocity of cells) changes with time in this model to mimic the transition between migration states. For the CTH model, we incorporate both cellular and temporal heterogeneity into the model by allowing all of the cells to undergo temporal transitions in migration states but in their own ways.

All of the theories except the HO model successfully reproduce the ensemble migration properties (〈(Δ*r*)^2^(*t*)〉 and *G*(*r*, *t*)) averaged over the cell population, which implies that one may employ either the cellular heterogeneity or the temporal heterogeneity to explain the average cell migration. However, when investigating the rescaled spatiotemporal correlation function *g*_*i*_(*r*, *t*) of individual cells, the CH model fails to reproduce rescaled *g*_*i*_(*r*, *t*) even qualitatively. Only the CTH model (with both cellular and temporal heterogeneity incorporated) reproduces the ensemble and individual spatiotemporal correlation functions of A549 cells successfully, which implies that both cellular and temporal heterogeneity together lead to anomalous cell migration.

Previous studies have reported that the temporal and/or cellular heterogeneity played critical roles in the cell migration of other cell lines^13,15,36^. The application of the CTH model to other cell lines should be a topic of interest. Previous studies focused mostly on the population averaged migration properties like 〈(Δ*r*)^2^(*t*)〉 and *G*(*r*, *t*), for which models with only temporal or cellular heterogeneity (in case of this study, the CH and TH models) worked well. One has to obtain and investigate the migration properties of single cells (such as *g*_*i*_(*r*, *t*)) when trying to study the effects of both temporal and cellular heterogeneity on the cell migration in more details. Beyond studies on population-averaged cell migration, the CTH model may serve as a framework for the migration properties of single cells.

## Materials and Methods

### Cell culture

An experiment was performed with A549 lung adenocarcinoma cancer cell line. Cancer cells were maintained in Dulbecco’s modified Eagle’s medium (DMEM) (ThermoFisher, MA, USA) supplemented with 10% fetal bovine serum (FBS) and 0.1 % gentamycin at 37 °C in a CO_2_ incubator. For real-time imaging, 5 × 104 cells, plated in the 60 *mm* cell culture plate, were monitored under an inverted time-lapse microscope (Lumascope 500, Etaluma, Carlsbad, USA) equipped with a 4× Olympus phase contrast objective. Time-lapse images (1280 × 800 pixel) were obtained every 2 minutes for 24 or 48 hours with Lumaview 500 software.

In order to exclude the possibility that heterogeneity in A549 cell migration would be caused by cross-contamination of other cell types with a different genetic background, we performed short tandem repeat (STR) DNA fingerprint analysis using 16 STR loci on the chromosomes through the Korean Cell Line Bank (KCLB). The A549 cells employed in this experiment exhibited identical genetic markers to the reference A549 cells (Table S1 in Supporting Information).

The substrate was often coated with extracellular matrix (ECM) proteins such as collagen and fibronectin to mimic the ECM *in vitro*, which is normally broken by proteases secreted from the cancer cells in the cellular environment. However, in this study, we attempted to compare to theoretical models for cell migration and minimize the unpredictable factors. We therefore used commercially available tissue culture plates with no further treatment.

A549 lung cancer cells (drug resistant with K-Ras mutation) are one of the typical lung adenocarcinoma cell models and are widely used in cancer biology because their epithelial characteristics are converted readily to mesenchymal characteristics through epithelial mesenchymal transition (EMT) due to their plasticity^56^. Considering the dramatic change in cellular characteristics during EMT, which is closely associated with not only cancer malignancy but also metastasis^57^, the development of a mathematical model for A549 cell movement would be important to further characterization of the movement of isogenic mesenchymal-type cancer cells^58^.

### Cell tracking

When analyzing the trajectories of cells, we consider A549 cells that are not divided for a 24 h measurement period. We obtain the trajectories of 212 A549 cells in our experiment. The center of the cell nucleus is determined to be the position of the cell. The initial positions (or the centers of the cell nuclei) of A549 cells were identified manually, after which the cell positions were obtained by an automatic tracking method via a weight-average of pixel positions as follows:

$${\hat{x}}_{i}=\frac{\int \,\int \,x{S}_{i}(x,y)dxdy}{\int \,\int \,{S}_{i}(x,y)dxdy},$$

where subscript *i* is a cell index, ${\hat{x}}_{i}$ is the estimated x position of the *i* th cell, and (*x*, *y*) is the position vector of a pixel in the image from time-lapse microscopy. In our cell images, 1 pixel unit is 5 *μ*m long. The score function (*S*_*i*_(*x*, *y*)) represents the probability that the position of the pixel would correspond to the position of the *i* th cell. The y position (${\hat{y}}_{i}$) of the *i* th cell can be estimated in the same way.

*S*_*i*_(*x*, *y*) consists of three functions of a pixel position (*x*, *y*), i.e., ${S}_{i}(x,y)={S}_{i}^{1}{S}_{i}^{2}{S}_{i}^{3}$. The first score function ${S}_{i}^{1}$ is used to identify the *i* th cell at a current position at time *t* with the *i* th cell with a previous position at time *t*−Δ*t*. Here, Δ*t* is the time resolution of the time-lapse microscopy in this study. Because Δ*t* = 2 min is much smaller than the characteristic timescale of the cell migration, there should be little difference in the cell position between two consecutive images. Therefore, pixels around the previous position of cells should get a high score, for which we suggest the following score function for ${S}_{i}^{1}$:

$${S}_{i}^{1}(x,y)=\frac{1}{{N}_{1}}{e}^{-\{(x-{x}_{i0}{)}^{2}+{(y-{y}_{i0})}^{2}\}/{\sigma }_{1}^{2}},$$

where (*x*_*i*0_, *y*_*i*0_) is the previous position of the *i* th cell at time *t*−Δ*t*, *N*_1_ is a normalized constant and *σ*_1_ = 12 pixels is a parameter used in this study. The second score function (${S}_{i}^{2}$) is employed to locate the center of the cell nucleus. In the cell image obtained by the time-lapse microscope equipped with a phase contrast objective, a pixel around the center of cell nucleus has a lower degree of brightness (*f*_*i*_(*x*, *y*)) than the pixels corresponding to the other parts of the cell (Fig. 1). Therefore, we define (${S}_{i}^{2}$) as follows:

$${S}_{i}^{2}(x,y)=\frac{1}{{N}_{2}}{e}^{-{\{{f}_{i}(x,y)\}}^{2}/{\sigma }_{2}^{2}},$$

where the degree of the brightness of a pixel *f*_*i*_(*x*, *y*) is rescaled from 0 to 100, *N*_2_ denotes a normalized constant and *σ*_2_ = 10 is a parameter for the brightness of pixels.

We introduce the third score function (${S}_{i}^{3}$) to exclude pixels outside a cell, i.e.,

$${S}_{i}^{3}(x,y)=(\begin{array}{cc}\mathrm{1,} & {\rm{inside}}\,{\rm{cell}}\,{\rm{area}},\\ \mathrm{0,} & {\rm{outside}}\,{\rm{cell}}\,{\rm{area}}.\,\end{array}$$

Here, we determine the pixel at position (*x*, *y*) to be outside the cell if *f*_*i*_(*x*, *y*) − *f*_*i*_(*x*_*i*0_, *y*_*i*0_) would be larger than a threshold value (=10) because a pixel around the cell surface is brighter than pixels corresponding to the other parts of the cell (Fig. 1). All three score functions are estimated only when the pixel position is within a cutoff length (=50 *μm*(10 pixels)) from the previous cell position. Note that the size of A549 cells ranges from 70 to 100 *μ*m. The representative trajectories of A549 cells are shown in Fig. 2(A).

In this study, we estimate and report the position of each cell by counting all of the certain digits in measurements plus the first uncertain digit. Because the estimated cell position is determined as the weighted average of pixel positions, the first uncertain digit corresponds to 1/10 pixel unit (0.5 *μ*m). (See the Supporting Information for details). We also report dynamic properties by setting the sampling time to 34 min even though time-lapse images were obtained every 2 minutes for 24 h. Two minutes is too short for a cell to migrate by more than a unit pixel (Fig. S2). Only after 34 min did the root of the mean-square displacement of A549 cells reach 5 *μ*m (1 pixel unit). As shown in Fig. S2, the mean-square displacement of cells is independent of the sampling time.

## Supplementary information

Supplementary Information
