# Supplementary material for: Stochastic and Heterogeneous Cancer Cell Migration: Experiment and Theory
Source: Sci Rep. 2019 Nov 8;9:16297. doi: 10.1038/s41598-019-52480-3 (PMC6841739; doi:10.1038/s41598-019-52480-3)
Supplement: Supplementary file 1 — Supplementary Information [file 41598_2019_52480_MOESM1_ESM.pdf]

Supporting Information for

Stochastic and Heterogeneous Cancer Cell Migration: Experiment and Theory

T. Kwon[1], O.-S. Kwon[2], H.-J. Cha[3]* and B. J. Sung[1]*

[1]Department of Chemistry, Sogang University,

Seoul 04107, Republic of Korea and

[2]Department of Life Sciences, Sogang University, Seoul 04107,

Republic of Korea

[3]College of Pharmacy, Seoul National University, Seoul 08826, Republic of Korea

I. THE SPATIAL PRECISION AND SAMPLING TIME OF THE EXPERIMENT

We take a track of A549 cells and estimate the positions of those cells by calculating the weighted average of pixel positions as discussed in the manuscript. Between two consecutive frames of snapshots, the estimated position of an individual A549 cells usually changes by less than 1 pixel unit. As shown in Figure S1 below, for example, the estimated cell position is the weighted average position of 9 gray pixels (with score functions equal to 1). One frame later in the Figure S1, the cell either changes its shape or migrates slightly such that the arrangement of gray pixels changes accordingly. We follow the conventional strategy to handle the uncertainty in measurements and count the significant figures: we report a measurement by recording all the certain digits in measurements plus the first uncertain digit. As shown in Figure S1, the first uncertain digit corresponds to 1/10 pixel unit, which corresponds to 0.5 μm.

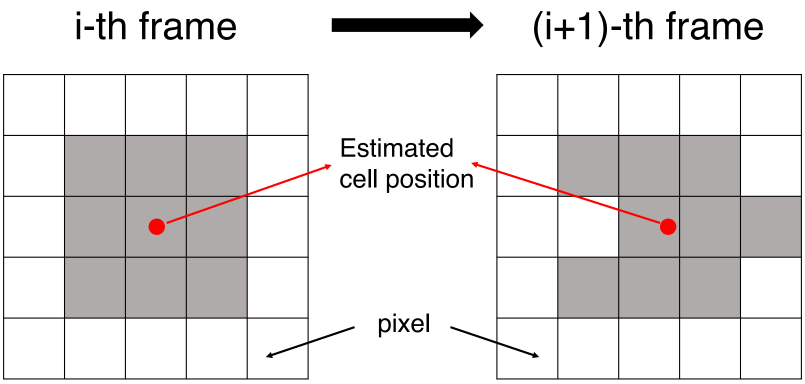

Figure S1. The schematic figure for the estimation of the cell position. Suppose that gray and white pixels have scores of 1 and 0, respectively. The cell position (red circle) is determined by the weight-average of pixel positions based on the score functions.

We change the sampling time in the analysis of the cell migration data from 2 min to 34 min. 2 min is too short for a sampling time because A549 cells hardly migrate during 2 min. Only after 34 min, A549 cells migrate on average by the size of one pixel. As shown below, the mean-square displacement ($\langle(\Delta\boldsymbol{r})^2(\boldsymbol{t})\rangle$) is hardly dependent on the sampling time. Only when t = 34 min, $\langle(\Delta\boldsymbol{r})^2(\boldsymbol{t})\rangle$ reaches 25 μm^2, which corresponds to one pixel unit. In our study, unless otherwise noted, we analyze all dynamic data by

using the sampling time of 34 min. Note that the sampling time of 34 min is still a short time scale in our experiment. Because the estimated size of A549 cells is about 70-100 μm, A549 cells migrate by about 1/20 of their own size during 34 min. The mean-square displacement of A549 cells is proportional to time after 200 min (as shown in Figure 2(B) and Figure S2), which is greater than the sampling time (34 min). Therefore, the sampling time of 34 min is enough to capture the persistent movement of A549 cells in our experiment. In order to estimate the localization error (σ_{err}), we also use the sampling time of 34 min and the equation 6 in the manuscript for the mean-square displacement ($\langle(\Delta \boldsymbol{r})^2(\boldsymbol{t})\rangle$). Even though we do not consider a dynamic behavior of cells at timescales shorter than 34 min (because we employ the sampling time of 34 min), we find that $\langle(\Delta \boldsymbol{r})^2(\boldsymbol{t})\rangle$ obtained from shorter sampling times (of 2 min and 6 min) do not change at long times as shown in Figure S2.

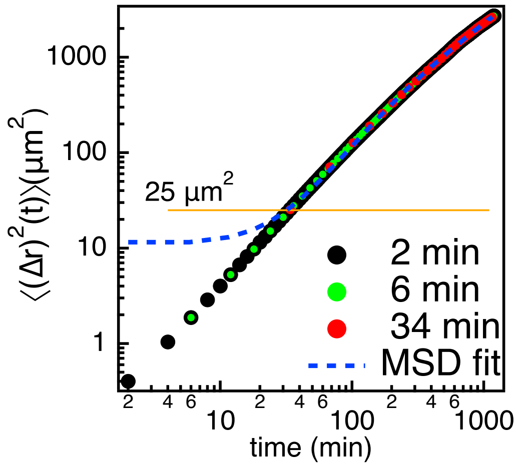

Figure S2. $\langle(\Delta \boldsymbol{r})^2(\boldsymbol{t})\rangle$ of A549 cells estimated by using various sampling time. A blue dotted line is a fitting function (Equation 6 in the manuscript) of MSD, for which a sampling time of 34 min is used. An orange line is a guide to 25 μm^2.

The fitting function of $\langle(\Delta \boldsymbol{r})^2(\boldsymbol{t})\rangle$ (blue dotted line) is obtained by fitting $\langle(\Delta \boldsymbol{r})^2(\boldsymbol{t})\rangle$ of A549 cells with the sampling time of 34 min (red circles). As shown in Figure S2, the fiting function (blue dotted line) is different from the experimental data obtained from shorter sampling times (black and green circles) before 34 min. The fitting function for the mean-square displacement in Figure S2 is based on the Equation 6, which

considers the localization error (σ_{err}) that arose due to the spatial resolution of our experiment. If σ_{err} were to be zero, the fitting function would be zero at time t = 0. As mentioned above, however, our spatial resolution of the experiment is limited to the pixel size (5 μm) such that the second term with σ_{err} in the Equation 6 for the mean-square displacement should lead to the seemingly biased fitting function. Note, however, that we are mainly concerned about the long time migration behavior of cells after 34 min. The fitting function of MSD is the same with $\langle(\Delta\boldsymbol{r})^2(\boldsymbol{t})\rangle$ of the HO model in Figure 2(B).

II. THE PERSISTENT TIME AND CELL SPEED OF A549 CELLS

Even though PRW model and CH model expect that the velocity autocorrelation function $\langle v_i(t)v_i(0)\rangle/\langle {v_i}^2(0)\rangle$ of an individual cell would be exponential. However, experimental results for $\langle v_i(t)v_i(0)\rangle/\langle {v_i}^2(0)\rangle$ of each cell (not from simulations) were often non-exponential but the parameters from the experimental trajectories have been still extracted for PRW models and other theories. There have been many reports where $\langle v_i(t)v_i(0)\rangle/\langle {v_i}^2(0)\rangle$ was not exponential. For example, ensemble-averaged $\langle v_i(t)v_i(0)\rangle/\langle {v_i}^2(0)\rangle$ of HT1080 cells was not exponential in both two and three dimensions (*Proc. Natl. Acad. Sci.* 111, 3949 (2014)). However, parameters such as persistent times were extracted from experiments based on PRW model and were re-used to describe the cell migration. In case of Dictyostelium cells (*Phys. Biol.* 8 046006 (2011)), for example, $\langle v_i(t)v_i(0)\rangle/\langle {v_i}^2(0)\rangle$ of each cell differed significantly for different cells, which indicates that the cell migration should be very heterogeneous. More importantly, $\langle v_i(t)v_i(0)\rangle/\langle {v_i}^2(0)\rangle$ of each Dictyostelium cell was not exponential. In this case, even if one were to obtain the ensemble-average of $\langle v_i(t)v_i(0)\rangle/\langle {v_i}^2(0)\rangle$, the ensemble-average of $\langle v_i(t)v_i(0)\rangle/\langle {v_i}^2(0)\rangle$ would not be exponential.

Such a non-exponential $\langle v_i(t)v_i(0)\rangle/\langle {v_i}^2(0)\rangle$ might be an indication that PRW model (HO model) would not be a proper way to describe the cell migration. However, in order to test the PRW model and CH model (that is based on PRW model), one still has to extract parameters from experiments, perform numerical simulations with the extracted parameters, and compare the numerical simulations with the experiments. One of the important parameters is the persistent time, which represents how long the trajectory of the cell would be persistent. In order to extract the representative value for the persistent time in this study, therefore, we estimate the time when $\langle v_i(t)v_i(0)\rangle/\langle {v_i}^2(0)\rangle = 1/e$ by using linear interpolation, which we determine as a persistent time (P_i) for each cell as shown in Figure S3. If each single A549 cell were to follow PRW model faithfully, the equation of $\langle v_i(t)v_i(0)\rangle/\langle {v_i}^2(0)\rangle = 1/e$ would provide the expected persistent time. However, $\langle v_i(t)v_i(0)\rangle/\langle {v_i}^2(0)\rangle$ of each A549 cell is not exponential like many other types of cells. But, we still need a persistent time for the single A549 cell to test CTH and CH models, for which we use $\langle v_i(t)v_i(0)\rangle/\langle {v_i}^2(0)\rangle = 1/e$ to extract the representative

value for the persistent time. Also, we obtain discrete values of P_{i}, which are multiples of 34 min (instead of continuous values obtained from linear interpolation). Our dynamic models need dynamic properties with the sampling time of 34 min to conduct stochastic simulations, such as spatiotemporal correlation functions of single cells at the persistent time ($g_i(r,t=P_i)$). Therefore, we need to perform stochastic simulations of dynamic models using discrete persistent times. The cell speed (S_i) is also obtained from the experiment by calculating the time-averaged magnitude of mean velocity of the ith A549 cell. Interestingly, CH model with such parameters reproduces the mean-square displacements (MSD) and the ensemble-averaged spatiotemporal correlation function (G(r,t)) even quantitatively well (Figure 2 in the manuscript). This indicates that our way of extracting persistent times from A549 cell trajectories should not be improper.

We show all the cell speed and persistent time of individual cells obtained from A549 cells (Figure S4). Note that the discrete values of P_i are multiples of 34 min because 34 min is the sampling time in this study in the analysis of the dynamic properties such as the cell velocity, $\langle(\Delta \boldsymbol{r})^2(\boldsymbol{t})\rangle$, and G(r,t).

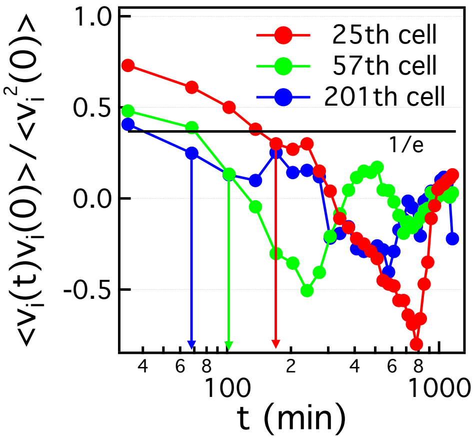

Figure S3. A few representative velocity autocorrelation functions. A black line is a guide to 1/e and arrows indicate corresponding persistent times.

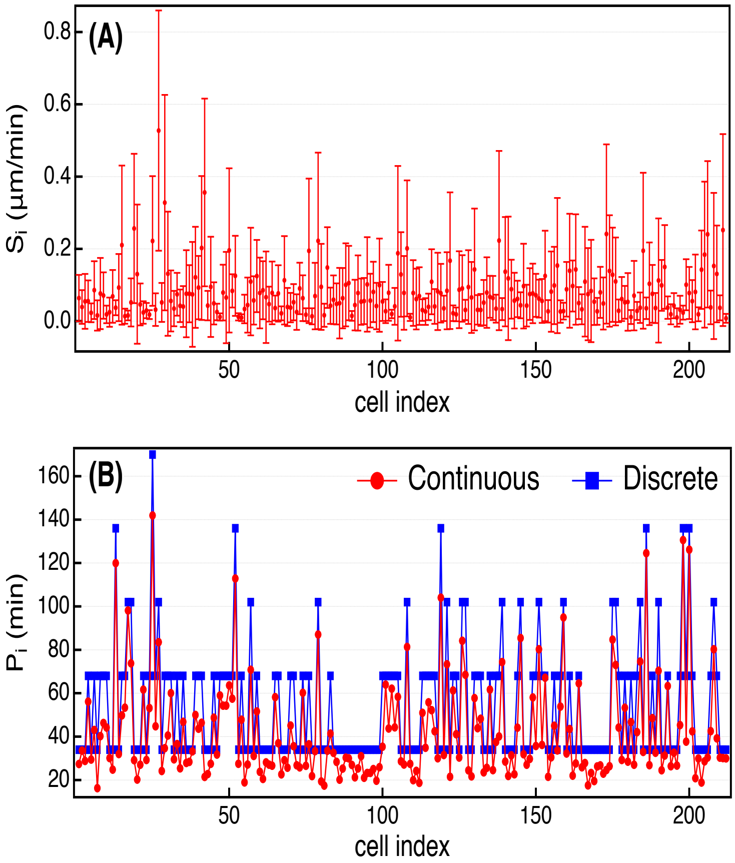

Figure S4. (A) Cell speed (S_i) and (B) continuous (red circles) and discrete (blue squares) persistent time (P_i) obtained from the velocity autocorrelation functions of individual A549 cells

III. EXAMPLES OF $\beta_i(t)$.

In TH model, $\beta(t)$ is sampled from $2\pi r$G(r,t = P) of the A549 cells, where P is the persistent time. That is, $\beta(t)$ is the magnitude of displacement during time t = P. Similarly, in CTH model, $\beta_i(t)$ is sampled from $2\pi r g_i$(r,t = P_i) of the ith A549 cell, where P_i is the persistent time of the ith cell. Unlike TH model, the distribution, from which the noise of each cell is sampled, differs for different cells. Figure S5 depicts examples of $\beta_i(t)$ sampled for the 14th and 42th cells.

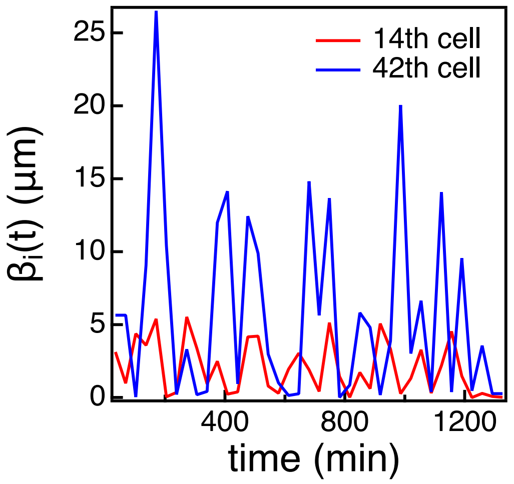

Figure S5. Examples of $\boldsymbol{\beta_i(t)}$. $\boldsymbol{\beta_i(t)}$ of the 14th cell (red) and the 42th cell (blue) as a function of time.

IV. THE PARAMETER A's IN TH AND CTH MODELS

TH and CTH models use fitting parameters, (A_{th} for TH model and A_{cth} for CTH model) as discussed in the manuscript. A's are parameters that indicate how much the cell migration would be persistent. We find the value of A's by comparing $\langle(\Delta \boldsymbol{r})^2(\boldsymbol{t})\rangle$ of A549 cells with simulation results of TH and CTH model at long times. As shown in the Figures R6(A) and (B), A_{th} = 2.5 and A_{cth} = 2.4 are the best fitting parameters that lead to a quantitative agreement in A_{cth} between the experiment and simulations.

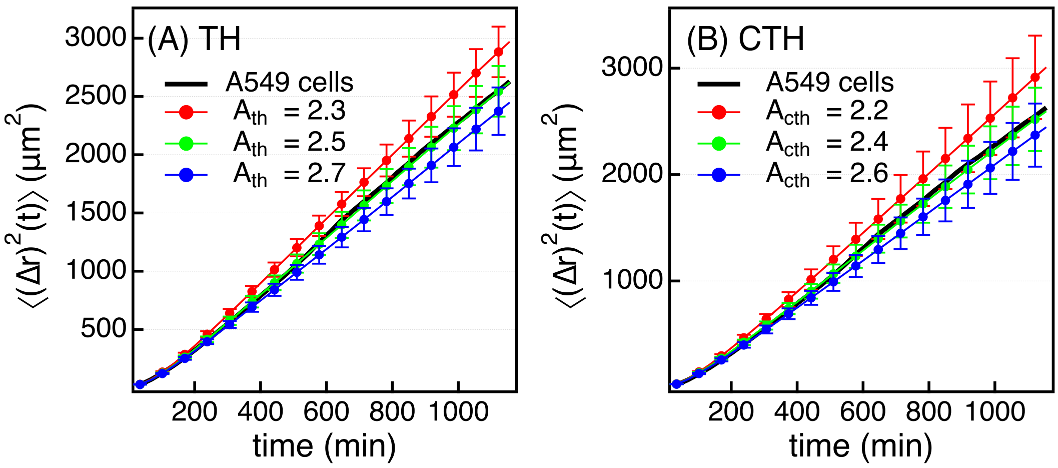

Figure S6. $\langle(\Delta \boldsymbol{r})^2(\boldsymbol{t})\rangle$ obtained from A549 cell trajectories (symbols) and simulation trajectories from (A) TH model and (B) CTH model.

V. THE SPATIALTEMPORAL CORRELATION FUNCTION OF INDIVIDUAL CELLS IN A549 CELLS AND FOUR MODELS

We calculate the spatiotemporal correlation functions ($g_i(r,t)$) at t = 408 min for some of the individual A549 cells in order to show how heterogeneous the cell migration of A549 cells is. As shown in Figure S7, $g_i(r,t)$ of the 25th cell spreads beyond r = 40 μm, implying that the 25th cell migrates by more than 40 μm during 408 min. On the other hand, other cells do not migrate as much as the 25th cell.

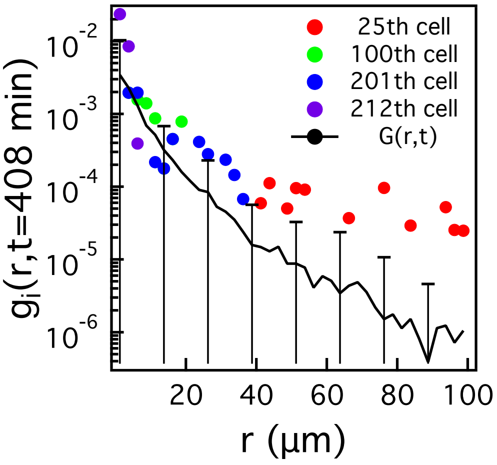

Figure S7. The spatiotemporal correlation functions of the 25th, 100th, 201th, and 212th cells. The black line represents the ensemble averaged spatiotemporal correlation function (G(r,t)) of A549 cells.

We also calculate $g_i(r,t)$ by using trajectories from four different models and parameters obtained from the 25th, 201th, and 212th cells. If a theory were to be successful, $g_i(r,t)$ obtained from the simulation with parameters for each A549 cell should be similar to $g_i(r,t)$ of the A549 cell. We also compare $g_i(r,t)$ to G(r,t), which is an ensemble average of $g_i(r,t)$ over all the simulation trajectories.

Figures S8(A) and (C) show that $g_i(r,t)$'s of the three cells in HO model and TH model collapse onto the ensemble averaged spatiotemporal correlation function (G(r,t)). This is because HO model and TH model do not incorporate the cellular heterogeneity. The 25th, 201th, and 212th cells have all similar $g_i(r,t)$'s unlike A549 cells in the experiment. On

the other hand, Figures S8(B) and (D) show that the cell-to-cell variation in $g_i(r,t)$'s is captured well in CH and CTH models. As in the experiment for A549 cells, the 25th cell migrates beyond 40 μm while others do not.

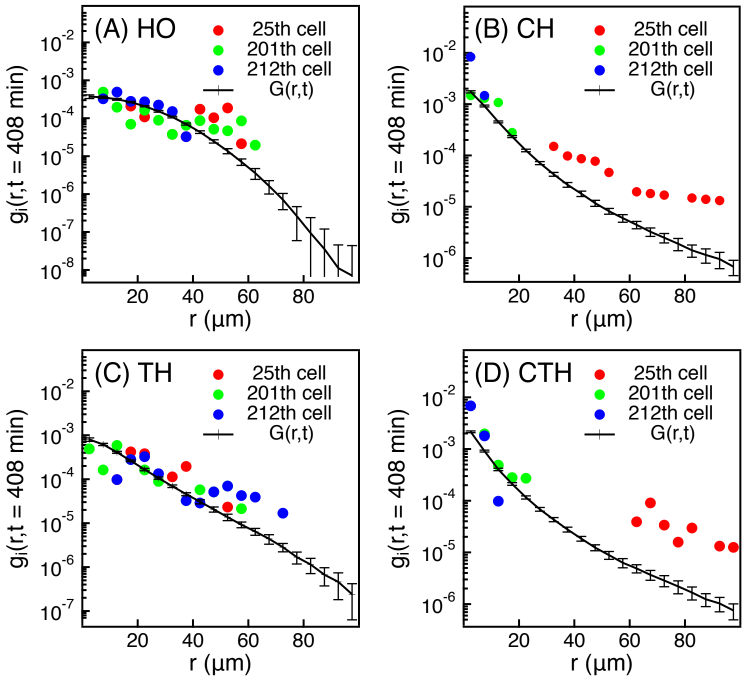

Figure S8. The spatiotemporal correlation functions $g_i(r,t)$ of the 25th, 201th and 212th cells at t = 408 min obtained from (A) HO model, (B) CH model, (C) TH model, and (D) CTH model. The black line represents the ensemble averaged spatiotemporal correlation function (G(r,t)) of each model.

VI. THE DISTRIBUTION OF PERSISTENT TIMES

The persistent time P_i of each A549 cell is estimated by calculating the velocity autocorrelation function of the A549 cell. Figure S9 depicts the distribution function ($\rho(P_i)$) of P of A549 cells. Figure S9 also depicts the distribution functions ($\rho(P_i)$) from stochastic simulations based on CH, TH and CTH models. Note that the stochastic simulations based on CH, TH and CTH models employ the persistent time as parameters for the simulations. We re-estimate the persistent time from the simulation trajectories in order to confirm that the stochastic simulations provide a proper persistent time distribution. Not surprisingly, $\rho(P_i)$'s from TH model are quite narrow while $\rho(P_i)$'s from CH and CTH models are broad like A549 cells. This is attributed to the fact that TH model does not take the cellular heterogeneity into account.

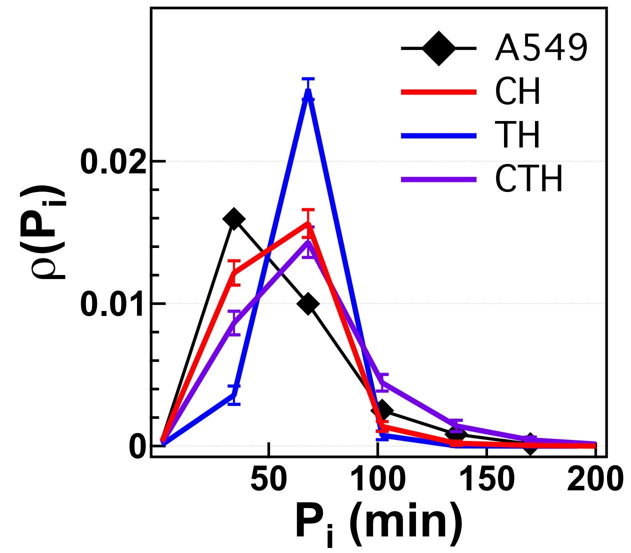

Figure S9. The distributions of persistent times of A549 cells (black symbols), CH model (red), TH model (blue), and CTH model (purple).

VII. THE MAGNITUDE OF NOISE AND THE MAGNITUDE OF MEAN DEVIATIONS OF ACCELERATION

In Figure S10, we compare the velocity dependence of noise of A549 cells and four models. We estimate the magnitude of the noise term as the magnitude of mean deviations of acceleration (Pedersen et al, *Phys. Rev. E* 94, 062401 (2016)). The acceleration $\vec{a}(t) = (\vec{v}(t+dt) - \vec{v}(t))/dt$ is calculated. And we consider the components of acceleration parallel and orthogonal to $\vec{v}(t)$ (that is to say, $a_p = \vec{a}(t) \cdot \vec{v}(t)/|\vec{v}(t)|$ and $a_{np} = \vec{a}(t) - \vec{a}(t) \cdot \vec{v}(t)/|\vec{v}(t)|$). We calculate the magnitude of mean deviations of acceleration, i.e. $|a_p - \langle a_p \rangle_v|$ and $|a_{np} - \langle a_{np} \rangle_v|$, where $\langle a_p \rangle_v$ and $\langle a_{np} \rangle_v$ are the conditional averaged components of acceleration at given $\vec{v}(t)$. The parallel component $|a_p - \langle a_p \rangle_v|$ is depicted and discussed in Figure 6 of the manuscript. Figure S10 depicts $|a_{np} - \langle a_{np} \rangle_v|$ as a function of the cell speed. Just like the parallel component, the magnitude of noise of HO model is uniform regardless of the speed. On the other hands, the magnitude of noise of A549 cells is different from the velocity dependence of HO model. Such velocity dependence of noise of A549 cells is reproduced by CH model, TH model, and CTH model qualitatively (Figure S10(B), (C), and (D)).

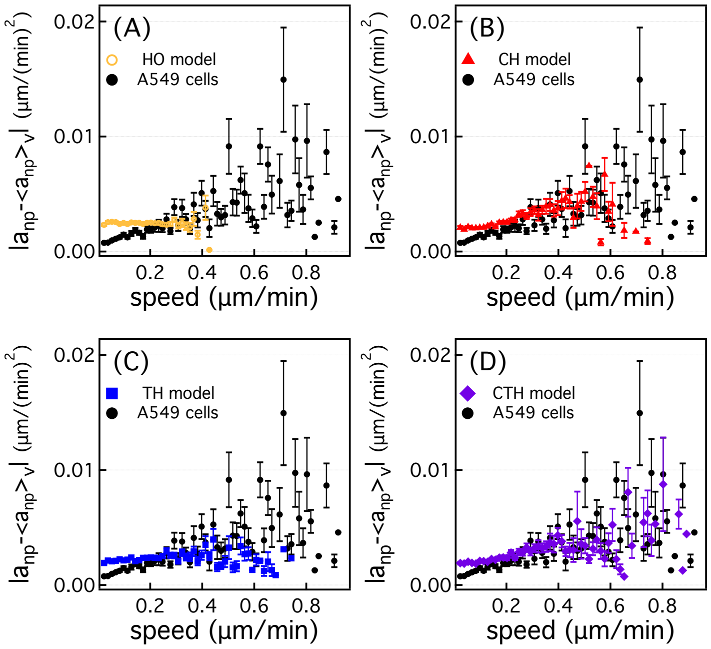

Figure S10. The magnitude of mean deviation ($|a_{np} - \langle a_{np} \rangle_v|$) of the component of acceleration orthogonal to $\vec{v}(t)$ versus speed. Black markers represent A549 cells. (A) Yellow markers represent HO model. (B) Red markers represent CH model. (C) Blue

markers represent TH model. (D) Purple markers represent CTH model. Error bars indicate the standard errors on the mean.

VIII. DNA FIGERPRINTING OF A549 CELLS.

We performed short tandem repeat (STR) DNA fingerprint analysis using 16 STR loci on the chromosomes through the Korean Cell Line Bank (KCLB) in order to investigate whether heterogeneity in A549 cell migration arose due to the cross-contamination of other cell types with a different genetic background or not. As shown below in Table S1, A549 cells in our experiment showed identical genetic markers compared with the reference cells.

Sample	A549 (Cont)	A549 (Exp)	Sample	A549 (Cont)	A549 (Exp)
D8S1179	13, 14	13, 14	D2S1338	24	24
D21S11	29	29	D19S433	13	13
D7S820	8, 11	8, 11	Vwa	14	14
CSF1PO	10, 12	10, 12	TPOX	8, 11	8, 11
D3S1358	16	16	D18S51	14, 17	14, 17
TH01	8, 9.3	8, 9.3	Amelogenin	X, Y	X, Y
D13S317	11	11	D5S818	11	11
D16S539	11, 12	11, 12	FGA	23	23

Table S1. STR DNA profile of experimental A549 cells (Exp) compared with reference A549 cells (Cont). The result of DNA fingerprinting at 16 STR loci is presented.
